# GSK-3β Allosteric Inhibition: A Dead End or a New Pharmacological Frontier?

**DOI:** 10.3390/ijms24087541

**Published:** 2023-04-19

**Authors:** Beatrice Balboni, Mirco Masi, Walter Rocchia, Stefania Girotto, Andrea Cavalli

**Affiliations:** 1 Computational and Chemical Biology, Istituto Italiano di Tecnologia, Via Morego 30, 16163 Genoa, Italy; balboni.beatrice@gmail.com (B.B.); mirco.masi@iit.it (M.M.); 2 Computational mOdelling of NanosCalE and bioPhysical sysTems (CONCEPT) Lab, Istituto Italiano di Tecnologia, Via Enrico Melen 83, 16152 Genoa, Italy; 3 Structural Biophysics and Translational Pharmacology Facility, Istituto Italiano di Tecnologia, Via Morego 30, 16163 Genoa, Italy

**Keywords:** drug discovery, cancer, Alzheimer’s disease, inhibitors, Pocketron

## Abstract

Most kinase inhibitors are designed to bind to highly homologous ATP-binding sites, which leads to promiscuity and possible off-target effects. Allostery is an alternative approach to pursuing selectivity. However, allostery is difficult to exploit due to the wide variety of underlying mechanisms and the potential involvement of long-range conformational effects that are difficult to pinpoint. GSK-3β is involved in several pathologies. This critical target has an ATP-binding site that is highly homologous with the orthosteric sites of other kinases. Unsurprisingly, there is also great similarity between the ATP-binding sites of GSK-3β and its isomer, which is not redundant and thus would benefit from selective inhibition. Allostery would also allow for a moderate and tunable inhibition, which is ideal for GSK-3β, because this target is involved in multiple pathways, some of which must be preserved. However, despite considerable research efforts, only one allosteric GSK-3β inhibitor has reached the clinic. Moreover, unlike other kinases, there are no X-ray structures of GSK-3β in complex with allosteric inhibitors in the PDB data bank. This review aims to summarize the state of the art in allosteric GSK-3β inhibitor investigations, highlighting the aspects that make this target challenging for an allosteric approach.

## 1. Introduction

Glycogen synthase kinase 3 beta (GSK-3β) is a multifunctional serine/threonine protein kinase (47 kDa). It is widely studied and characterized for its involvement in many fundamental cellular pathways, where it interacts with more than 100 different substrates [1]. GSK-3β’s structure was first identified in 1980 [2], with many groups later contributing to its crystallization and structure definition. To date, the Protein Data Bank (PDB) contains about 92 X-ray and nuclear magnetic resonance (NMR) structures of GSK-3β in complex with a variety of inhibitors and co-factors. GSK-3β’s activity is finely regulated via its differential phosphorylation on Tyr216 (active form) and Ser9 (inactive form). While GSK-3β is constitutively active in cells, its Ser9 phosphorylation plays a role in maintaining homeostasis in response to intracellular and extracellular stimuli [1]. Initially identified as a key player in the insulin and Wnt/β-catenin pathways, GSK-3β also plays a central role in mitochondrial activity and metabolism [3], as well as in many other PI3K/Akt-mediated physiological pathways, such as Hedgehog, Notch, growth factors, and neurotrophins [4].

In recent years, there has been growing medical and pharmacological interest in this protein. Indeed, the altered expression and activity of GSK-3β and the impairment of its negative regulation are correlated with the development and progression of several pathologies. These include type II diabetes mellitus, immunological and chronic inflammatory diseases, different neurological pathologies characterized by cognitive deficits, and several types of cancer [5]. Specifically, dysregulation of GSK-3β activity has been reported in neurological diseases such as bipolar disorder (BD) [6,7,8] and in neurodegenerative disorders such as Alzheimer’s disease (AD) [9,10], Parkinson’s disease (PD) [11,12,13] and Huntington’s disease (HD) [14,15,16]. Overexpression and aberrant activation of GSK-3β are correlated with tau hyperphosphorylation (with its consequent dissociation from microtubules and aggregation into neurofibrillary tangles, NFTs), alteration of amyloid precursor protein (APP) processing and amyloid-β (Aβ)-induced neurotoxicity in AD, and increased α-synuclein expression in PD [17].

In addition to its role in suppressing tumor development and progression, GSK-3β is also pivotal to several pro-oncogenic pathways (e.g., Wnt/β-catenin-, Hedgehog-, Notch- and c-myc-mediated signaling) [18]. There is increasing evidence that the aberrant activity and expression of GSK3β promote the progression of various cancer types and their resistance to pharmacological or radiological treatments [19,20,21,22,23]. Indeed, GSK-3β’s tumor-promoting functions have been reported in 25 different cancer types. The most investigated cancer types in this context include cancers of the colon, rectum, pancreas, prostate, breast, and ovaries, glioblastoma, and leukemia [24,25]. The pro-oncogenic activity of deregulated GSK-3β sustains cancer cell survival, proliferation, migration, and invasion by suppressing or enhancing a broad range of molecular pathways (reviewed in [24]). However, GSK-3β’s role in cancer development is still debated, since GSK-3β may act as tumor suppressor or tumor promoter and, depending on the cancer type, the cancer development could be linked to GSK-3β’s overexpression or to its downregulation [26,27,28,29].

The literature indicates that GSK-3β inhibitors can reduce tau phosphorylation and tau toxicity [17], and that GSK-3β inhibition is a potential strategy for treating cancer [24]. Due to this involvement in a variety of diseases, GSK-3β inhibitors are needed. Indeed, many GSK-3β inhibitors are the subject of patents and/or clinical and preclinical investigations [30,31,32,33,34].

However, GSK-3β’s different roles in different pathologies mean it is challenging to rationally design GSK-3β inhibitors and develop them for therapeutic applications. To date, most GSK-3β inhibitors target the ATP-binding site. Due to high homology with most other kinases, this can lead to dangerous cross-activity effects (i.e., inhibition of other kinases). However, GSK-3 has two isoenzymes GSK-3α and GSK-3β (51 and 47 kDa, respectively) [35] that share 98% sequence identity in their kinase domains and more than 95% sequence identity at the catalytic site [36]. Despite displaying similar biological functions and expression patterns in many tissues, knock-out studies have shown that GSK-3α and GSK-3β are not completely redundant [35]. Indeed, the literature suggests that isoform-specific inhibitors may lead to specific therapies [23]. Despite their considerable sequence homology, GSK-3α and GSK-3β only share 36% identity in their C-terminal regions. Moreover, GSK-3α features an additional glycine-rich extension at its N-terminus [36], where more selective motifs may be envisaged.

In addition to the poor selectivity associated with targeting the ATP-binding site, another challenge is GSK-3β’s involvement in a variety of cellular pathways. Indeed, completely inhibiting its activity could simultaneously abrogate several signaling pathways, including essential pathways unrelated to the pathology, thereby leading to major adverse effects.

Various approaches have been proposed to improve selective GSK-3β inhibition. One approach is to identify druggable pockets outside the ATP-binding site. By binding to these alternative pockets, allosteric compounds may induce conformational changes in GSK-3β that affect its aberrant activity without directly competing with the ATP-binding site. Indeed, in principle, allosteric inhibitors offer an ideal way to limit side effects, because they should not have the promiscuity associated with ATP inhibition and should not inactivate nonpathological pathways involving GSK-3β.

Hence, this review aims to summarize the most recent insights into small-molecule allosteric GSK-3β inhibitors. Our key questions are whether this approach is suitable for the challenges posed by GSK-3β and whether enough progress has been made in the field, given the scale of research efforts.

## 2. GSK-3β Inhibition

**Figure 1:**
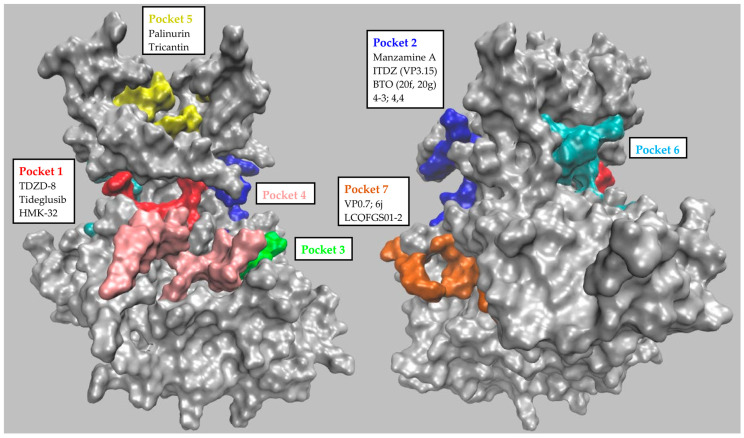
Representation of the main GSK-3β pockets, as described in Palomo et al., 2011 [37]. The left and right panels contain the front and rear views, respectively.

In the search for novel druggable and possibly allosteric sites on GSK-3β, Palomo et al. in 2011 were some of the first to publish a comprehensive and representative computational analysis [37]. They used a free geometry-based pocket identification algorithm (fpocket) complemented by a second tool (hpocket) that was able to apply the analysis to homologous proteins too. They applied these tools to 25 available GSK-3β structures, thus identifying seven well-conserved and likely druggable pockets. Three of these binding sites were the well-known ATP-binding site (Pocket 1, Figure 1), the substrate-binding site (Pocket 2, Figure 1), and the axin/fratide binding site (Pocket 3, Figure 1). The additional four binding pockets were potential allosteric sites located at the protein’s C-terminal lobe (Pocket 4 and 7, Figure 1), at the hinge region between the N- and C-terminal lobes (Pocket 6, Figure 1), and at the N-terminal lobe (Pocket 5, Figure 1). Table 1 reports the identity of the key residues that characterize the seven pockets identified by Palomo et al. This computational approach allowed the systematic classification and analysis of the available allosteric GSK-3β inhibitors and of those synthesized subsequently.

**Figure 2:**
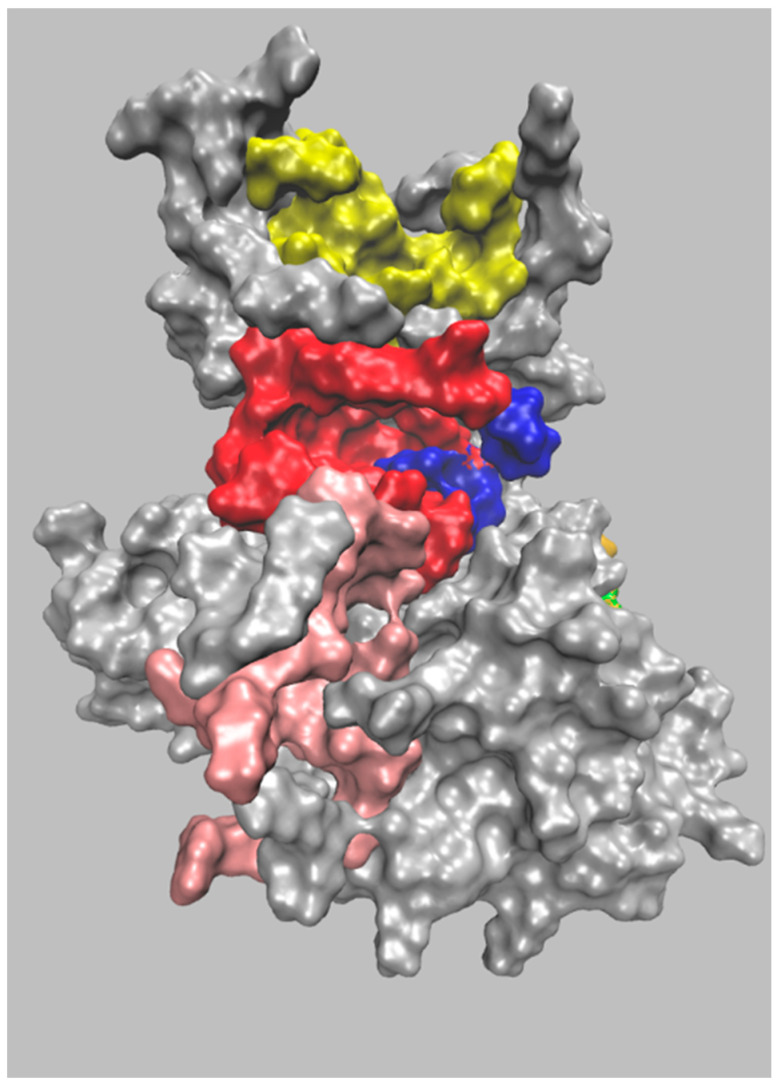
Front view representation of GSK-3β, with the main pockets found by Pocketron having the same colors as the closest pockets in Palomo et al., 2011 [37] (left panel of Figure 1). Note that the pockets are located in the same regions, although their shapes differ.

More recently, we used Pocketron tool (from the BiKi Life Science Software Suite 1.3.5), to corroborate and further substantiate the GSK-3β pockets analysis. Pocketron is a molecular dynamics (MD)-based pocket detection and analysis algorithm that can characterize the pockets occurring along a MD trajectory. Its analysis provides the geometric features of the identified pockets, particularly the volume and surface area, and points to potential surface-mediated allosteric connections by defining the crosstalk network between nearby surface regions [38]. In particular, we ran and analyzed a 1-µs-long MD simulation on the apo structure of GSK-3β. The reference structure was chain B of the system, with PDB code 1J1C, which was selected among the other available structures for its good resolution, wide sequence coverage, and above all, because it represents a structure obtained in absence of ligands and therefore devoid of the fingerprint of the binders. Pocketron uses NanoShaper v0.7 software to identify pockets in each input frame extracted from the trajectory and creates a timeline of the observed pockets [39,40]. By construction, many superficial cavities are found, which must be further filtered and validated in terms of their geometric and chemical features in order to estimate their ligandability. As was recently demonstrated, volume is a primitive but predictive indicator [41]. In our analysis, pockets with greater volumes were considered to be more relevant. As shown in Figure 2, our analysis substantially confirmed the presence of the main pockets described by Palomo et al., albeit with slightly different shapes and volumes.

The key residues of the main pockets identified by Pocketron (PDB 1J1C B numbering), related to those identified by Palomo et al. (residues in common are underlined), are reported below.

Pocket 1 in Palomo et al.: Ile540, Gly541, Asn542, Gly543, Val548, Gln550, Ala561, Lys563, Glu575, Met579, Val588, Leu610, Asp611, Tyr612, Val613, Pro614, Glu615, Thr616, Arg619, Lys661, Gln663, Asn664, Leu666, Cys677, Phe679, Gly680.

Pocket 2 in Palomo et al.: Ser544, Phe545, Asp659, Asp678, Gly680, Ser681.

Pocket 3 in Palomo et al.: Arg687, Ala702, Pro703, Phe707, Gly708, Ala709, Thr710, Asp711, Tyr712, Arg760, Glu761, Met762, Asn763, Pro764, Tyr801.

Pocket 5 in Palomo et al.: Met504, Val506, Lys514, Thr516, Tyr534, Thr537, Tyr549, Ile562, Lys564, Phe594, Ser596, Ser597, Gly598, Glu599, Asn607, Tyr605.

Pocket 6 in Palomo et al.: Gln524, Met579, Leu582, Asp583, Hie584, Ile587, Val588, Arg589, Leu590.

Pocket 7 in Palomo et al.: Ala648, Hie651, Ser652, Leu807, Thr808, Leu810, Gln843, Leu845.

Contrary to previous pockets, which are similar to the ones reported by Palomo et al., Pocket 4 is characterized as a canyon-like area, which was assigned to four smaller and partially overlapping putative pockets by Pocketron.

The key residues that characterize the four pockets are Ala621, Tyr624, Ser625, Glu627, Gln629, Thr630, Leu631, Leu725, Ala726, Leu729, Leu730, Phe783; Thr630, Val633, Glu727, Leu728, Phe783, Arg786; Tyr618, Ala621, Pro662, Leu725, Glu727, Leu730; and Tyr618, Pro662, Gln663, Ile695, Cys696, Ser697, Arg698, Tyr699, Leu725, Gly731.

**Figure 3:**
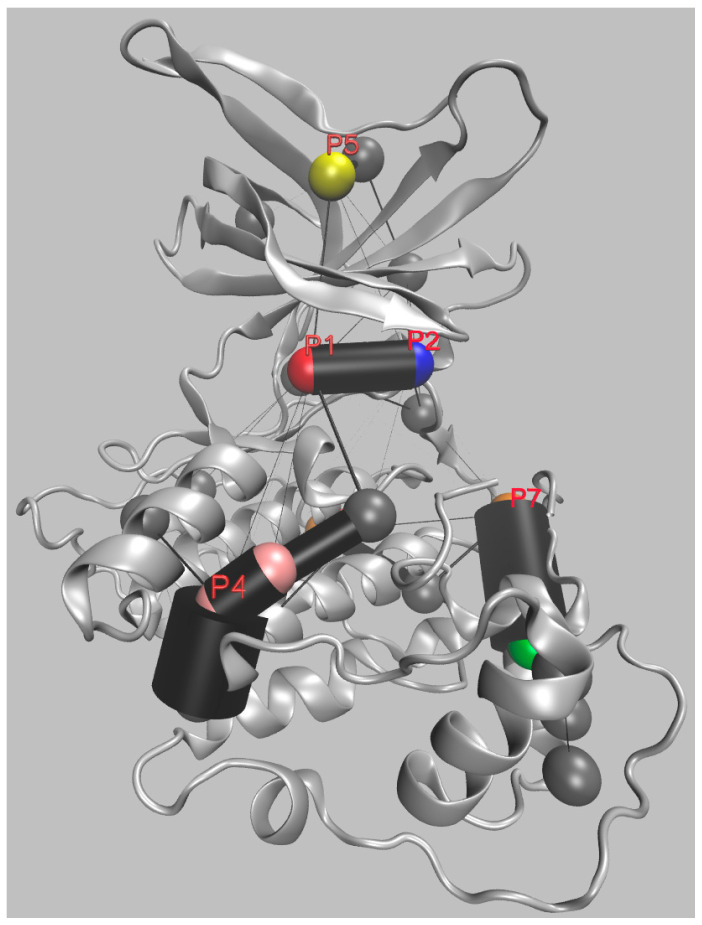
Front view of GSK-3β, with the main pockets (indicated as P1, P2, P4, P5 and P7) found by Pocketron represented as spheres. The spheres are color-coded, as in Figure 2, when located in the same region as the pockets identified by Palomo et al. Otherwise, the spheres are in gray or green. Black cylinders connect the pockets involved in crosstalk, with a radius proportional to the frequency of the crosstalk.

As expected, Pocketron also proposed other possible pockets. Figure 3 contains a different representation of the pockets found by Pocketron in GSK-3β. In Figure 3, each pocket is depicted as a sphere. The spheres are color-coded, as in Figure 2, when they correspond to a previously described pocket, and are otherwise left in gray.

The pockets involved in crosstalk at the protein surface are connected by a cylinder whose radius is proportional to the frequency of the crosstalk.

Notably, there is great crosstalk between pockets P1 (Palomo et al., numbering) and P2, confirming the tight connection between the orthosteric and substrate-binding pockets. Similarly, pocket P7 (a potential allosteric pocket) is clearly connected via extended crosstalk with an additional pocket (represented as a green sphere and characterized by the following key residues: Leu685, Val686, Arg687, Gly688, Glu689, Pro690, Asn691, Gly708, Ala709, Thr710, Asp711, Tyr712) that was not previously considered. Interestingly, pocket P1 (the orthosteric site) is also connected to a region of four highly interconverting putative pockets located in the region of pocket P4, which may be particularly interesting for drug discovery.

The additional pockets proposed by Pocketron (grey spheres), which could not be paired with any of those indicated in the work of Palomo et al., should also be investigated because they may contain allosteric sites with relevance for drug discovery.

### 2.1. Compounds and State of the Art

**Figure 4:**
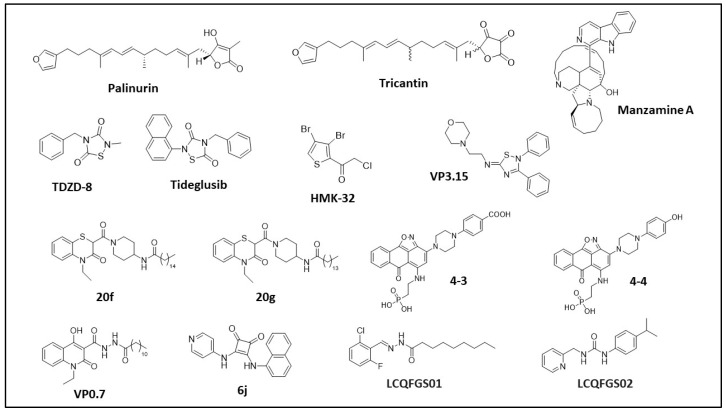
Structures of the available allosteric and non-ATP-competitive GSK-3β inhibitors.

Almost all the allosteric compounds reported to target GSK-3β are either natural products or molecules of synthetic origin. Computational docking analysis has confirmed all of them as binders of one of the allosteric pockets identified by Palomo et al., and corroborated by Pocketron. Table 2 and Figure 4 summarize the structures of the known allosteric and non-ATP-competitive GSK-3β inhibitors together with their binding parameters and putative binding pockets. Below, we briefly describe the distinguishing features of each inhibitor’s scaffold.

#### 2.1.1. Natural Compounds and Derivatives

##### Palinurin and Tricantin

In 2005, Alonso, D. et al., were the first to isolate and identify novel furanoterpenoid compounds from the Mediterranean sponges *Ircinia dendroides*, *Ircinia variabilis*, and *Ircinia oros* [42]. Through fractionation and purification of sponge extracts, followed by GSK-3β inhibition assays, they identified *Palinurin* and *Tricantin* furanosesquiterpenoids as new GSK-3β inhibitors (Patent WO 2005/054221 A1 Alonso et al.) with IC_50_ values of 4.5 μM and 7.5 μM, respectively [43]. These compounds showed good permeability to cell membranes and good ability to reduce tau phosphorylation in cell cultures [44]. Palinurin was first resynthesized by Pérez et al. in 2009 [45].

More recently, Martinez et al., used palinurin as a reference compound in pharmacophore and pharmacodynamics studies in order to elucidate its mechanism of action and identify potential novel GSK-3β inhibitors in a computational approach [46]. Palinurin is neither ATP-competitive nor substrate-competitive, and was first confirmed in 2013 as an allosteric inhibitor of GSK-3β [47]. Palinurin’s proposed binding mode to GSK-3β and its novel mechanism of action [47] were determined by using the CMIP program to select the best docking solutions for palinurin in the GSK-3β pockets previously identified by Palomo et al. [37,48]. The results showed four populated sites that were suitable for accommodating palinurin’s tetronic ring. The most populated solution was the ATP-binding site, but this was not considered due to palinurin’s allosteric behavior. The second most populated solution was the pocket at the N-terminal lobe at the top of the glycine-rich loop (pocket 5, Figure 1), i.e., the assigned binding site. Further molecular dynamic studies were performed to clarify palinurin’s inhibition mechanism and its key interactions with the protein. It was observed that when binding to GSK-3β, palinurin may actually affect the conformational flexibility of the glycine-rich loop, altering the accessibility of the γ-phosphate of ATP. To confirm this model, the Bidon–Chanal group compared the structure and activity of palinurin with several analogs (i.e., ascorbic acid derivatives and ircinins), further demonstrating that the compound’s amphipathic character is essential to achieving GSK-3β binding and activity inhibition [47].

##### Manzamine Alkaloids

In 1986, Manzamine A, a β-carboline alkaloid with a complex polycyclic system, was isolated and identified from the Okinawan sponge of the genus *Haliclona* [49,50]. The activity of this new class of compounds was initially characterized in 2006 by Rao et al., who reported a 70% GSK-3β inhibition at 25 μM [51]. Further pharmacokinetics and SAR studies showed that the entire Manzamine A structure was required to achieve GSK-3β inhibition, with its chemical precursors ircinal A and carboline being ineffective [52].

This study was the first to use kinetics analyses to confirm that Manzamine A is a non-ATP-competitive inhibitor. Manzamine A’s novel inhibition mechanism, initially proposed for GSK-3β, was later supported by cross-activity analyses of a panel of selected kinases. Manzamine A inhibits GSK-3β (IC_50_ = 10 μM), but also cyclin-dependent kinase 5 (CDK-5), with an IC_50_ value of 1.5 μM. The compound was also tested in vitro on SH-SY5Y human neuroblastoma cells, in which it decreased levels of tau phosphorylation. This was a promising starting point for potentially using Manzamine A to treat diseases involving GSK-3β, beginning with AD. Upon GSK-3β inhibition in different colorectal cancer (CRC) cell lines, Manzamine A induced cell cycle arrest, mitochondria-driven apoptotic cell death, and epithelial–mesenchymal transition (EMT) abolishment [53]. Moreover, in vitro treatment with Manzamine A reduced the metastatic potential of AsPC-1 pancreatic cancer cells and sensitized them to TNF-related apoptosis-inducing ligand (TRAIL)-induced apoptosis via GSK-3β blockade [54], suggesting that GSK-3β inhibitors may be effectively combined with TRAIL to treat pancreatic cancer [55]. The binding epitope of Manzamine A on GSK-3β and its binding conformations have also been investigated with a biocomputational approach [37,56,57]. Starting from the molecular docking of Manzamine A on a GSK-3β structure available in the PDB, the Osolodkin group used molecular dynamics studies with Autodock 4.0.1 software to analyze the different poses of Manzamine A on GSK-3β. The various docking solutions were clustered and used to define the most populated binding sites for Manzamine A on GSK-3β. Five sites were selected, and simulations of binding energies were performed using the MM-PBSA/GBSA method. Since the MD method did not provide sufficient information to identify the binding site for Manzamine A, several analytical methods were applied (i.e., conformational analysis of the ligand, visual analysis of the MD trajectories and the correlation maps). According to pocket ranking based on these approaches, site II (cavity between glycine-rich loop, loop C, and the activation loop, pocket 2, Figure 1) was selected as the potential binding site for Manzamine A. In support of these hypotheses, some residues in the identified pocket were further recognized as key residues in the kinase–substrate interaction [57], together with the Palomo group’s biocomputational and pharmacokinetic analyses [37], which confirmed pocket 2 as the binding site for Manzamine A. Finally, another group proposed a computational biology screening method to validate Manzamine A derivatives’ binding to GSK-3β [56]. Peng et al., used their docking software to confirm the substrate pocket (pocket 2) as the most probable binding site for Manzamine A.

#### 2.1.2. Synthetic Compounds and Derivatives

##### Thiadiazolidinones and Derivatives (TDZD)

In 2002, a novel family of non-ATP competitive GSK-3β inhibitors was identified [58] among a series of compounds previously used as efficient potassium channel openers [59] and cholinergic drugs [60]. Of these, TDZD-8 was particularly interesting because of its selectivity towards GSK-3β relative to other kinases (IC_50_ 2 μM for GSK-3β and >100 μM for CDK-1, casein kinase II (CK-II), protein kinase A (PKA), protein kinase C(PKC)), and because of its allosteric behavior (no competition with ATP or substrate). The crystal structure of TDZD-8 bound to GSK-3β has not yet been solved. However, by comparing SARs of different derivatives, researchers have proposed a putative binding site for TDZD-8 and its mechanism of action. Data suggest that TDZD-8 binds closely to residues Arg96, Tyr216, and Lys205 of GSK-3β, and that its inhibitory activity is tightly connected to the aromatic ring in N4 and the methyl moiety in N2. Moreover, it has been suggested that the heterocycle’s negative charge drives the recognition of the protein’s oxyanion site.

TDZD-8 is often used to inhibit GSK-3β in cellular and animal models and to induce pathology [61,62]. In 2017, TDZD-8 was shown to reduce hypoxic-ischemic brain injuries in mice [63]. Notably, TDZD-8-mediated GSK-3β inhibition induced cell death and reduced the growth of different tumor types, including colorectal cancer [64], leukemia [65], glioblastoma [66], prostate cancer [67], and ovarian cancer [68]. Moreover, TDZD-8′s blockade of GSK-3β exerted positive effects in AD and PD by preventing pro-apoptotic signaling cascades associated with neurodegenerative diseases [69], thereby improving motor function [70] and cognitive function [71,72], reversing α-synuclein increase and hyperphosphorylated tau accumulation [73], and disrupting the GSK-3β-regulated Kelch-like ECH-associated protein 1 (Keap1)–nuclear factor erythroid 2-related factor 2 (Nrf2) axis [74]. TDZD-8-mediated GSK-3β inhibition has been investigated in other pathological contexts, including type II diabetes mellitus [75] and various inflammatory diseases [76,77], suggesting that GSK-3β inhibition via TDZD-8 could be an effective therapeutic strategy for a broad range of pathologies.

Another compound in the TDZD family is Tideglusib (NP-12; NP031112), which was initially investigated as a promising non-ATP-competitive GSK-3 inhibitor. Serenò et al., first selected this compound in an in vivo study using transgenic mice as AD models [78]. Treatment with Tideglusib improved cognitive deficit and decreased tau phosphorylation, amyloid deposition, and neural loss. Tideglusib’s mechanism of action was further characterized by Domìnguez et al. in 2012, via ATP competitive assays associated with pharmacokinetics analyses of the dissociation rate and protein turnover analysis [79]. These revealed that Tideglusib is an irreversible non-ATP-competitive GSK-3β inhibitor, with the non-ATP-competitive property ascribed to the covalent irreversible modification of Cys199 in the GSK-3β active site. The crystal structure of the GSK-3β-Tideglusib complex has not yet been solved, and the exact binding mechanism is yet to be elucidated. Notably, Tideglusib is one of the few compounds currently undergoing clinical trials for AD [80,81] and supranuclear palsy [82,83] (https://clinicaltrials.gov, accessed on 20 February 2023). However, there is not yet sufficient evidence to support or reject a benefit of Tideglusib for AD. Additional clinical trials are required to investigate this compound’s effects on AD patients.

Tideglusib’s structure was also used in a computational study to identify new GSK-3β inhibitors [84]. A pharmacophore-based virtual screening of a library of compounds was pursued using Tideglusib moieties as reference. The researchers selected 416 molecules with pharmacophore features similar to those of similar Tideglusib. These molecules were then used in virtual screening on GSK-3β with Arguslab v4.0.1 software. Relative to Tideglusib, one compound (ZINC4192390) showed greater binding affinity for GSK-3β, which is likely because it more stably interacts with Cys199 at the entrance to the active site.

##### Halomethylketones (HMKs) and Derivatives

Halomethylketones (HMKs) are another family of synthetic non-ATP-competitive compounds. They were first described as GSK-3β inhibitors by Conde et al., in 2003. The Conde group investigated a library of α-methylketones, using various kinetics experiments to identify their putative IC_50_ values and their mechanism of GSK-3β inhibition [85]. Further SAR studies published in 2009 and 2011 [86,87] suggested that these compounds irreversibly bind GSK-3β by forming a covalent sulfur–carbon bond between their HMK moiety and residue Cys199 at the entrance to the active site. This modification is thought to modulate GSK-3β’s phosphorylation activity and thus change the correct position of ATP in the ATP-binding site. This hypothesis was supported by in silico models (i.e., *rDock*) [87]. In vitro biochemical studies with selected compounds of this family showed evidence of decreased tau phosphorylation and neurite outgrowth, with IC_50_ values in the low micromolar range [86]. Moreover, the selected HMKs did not significantly inhibit other kinases. These data confirmed the specificity of the HMK moiety binding to only Cys199 of GSK-3β. Crystal structures of the complex GSK-3β-HMK- are not yet available.

##### 5-Imino-1,2,4-Thiadiazoles (ITDZs)

Palomo et al. described -imino-1,2,4-thiadiazoles (ITDZs), another family of GSK-3β non-ATP-competitive inhibitors [88], as an optimization of the TDZD scaffold [58]. Kinetic competitive assays of the ITDZ derivatives showed that they to inhibit GSK-3β by more than 60%, with IC_50_ values in the low micromolar/submicromolar range [88]. In contrast to TDZDs, ITDZs are not ATP-competitive, but they do seem to be competitive with the GSK-3β substrate. To better characterize this compound family’s mechanism of inhibition, some were selected and used for computational docking studies. In silico tests suggested that ITDZs’ mechanism of inhibition may be linked to the 5-imino-1,2,4-thiadiazole scaffold. This scaffold can maintain the hydrogen bond interaction between the proton of its imino-charged group and Phe67 of GSK-3β. It can also maintain the aromatic S-π interaction between its thiadiazole heterocycle and the aromatic ring of Phe67 [88]. Palomo et al. also performed in vitro analyses of cellular models, which showed that these compounds reduce inflammatory activation and trigger neural differentiation in neural stem cells. Twenty of these derivatives were able to cross the blood–brain barrier (BBB) and were thus good candidates for further development into drugs for neurological diseases [88]. Recently, one of these derivatives (VP3.15) was tested in in vivo studies in models of retinitis pigmentosa, autoimmune encephalomyelitis (AE), and multiple sclerosis (MS) [89,90]. VP3.15 exerted neuroprotective, anti-inflammatory, and remyelinating effects in an MS mouse model [91], suggesting its potential as a disease-modifying drug for MS treatment. In addition to this promising pharmacological application, VP3.15 is the first dual GSK-3 cAMP phosphodiesterase 7 (PDE7) inhibitor ever reported [92].

##### Benzothiazinones (BTOs) and Derivatives

In 2012, researchers used virtual screening methods and experimental analyses to describe benzothiazinones (BTOs), another group of non-ATP-competitive GSK-3β inhibitors. Initially, virtual screening studies were performed with Autodock 3.0.5 and the Lamarckian genetic algorithm (LGA) in order to optimize a scaffold for the SAR analyses of BTZ (benzothiazepinone) derivatives [93]. This algorithm was selected because it allowed the researchers to consider a wide range of flexible ligand conformations within a rigid protein. A total of 150,000 drug-like molecules were screened, and twenty were selected for their binding affinities (binding free energy). Of these, BTZs were the most represented, with an interesting new scaffold. A computational analysis of these promising compounds highlighted the importance of substituents at the nitrogen atom. Indeed, the methyl group attached to the nitrogen atom was critical to improving binding affinities. In parallel, promising allosteric compounds were tested in GSK-3β inhibition and selectivity assays to describe the SAR of each substituent moiety [94]. Compounds with IC_50_ values in the micromolar range were suggested to have a putative allosteric binding mode (computational tool GOLD 5.0 from the Cambridge Crystallographic Data Center, Cambridge, UK). Hit compound **6V** was located between residues Arg209 and Ser236 (pocket 7, Figure 1), with its BTZ ring bound to Arg209 by a cation-π interaction.

More recently, the same group published interesting new molecules obtained from the evolution of previously published hits [95]. Compound BTO-5h is not competitive with ATP or the substrate, and has an IC_50_ value of 8 μM. In contrast, BTO-5s is substrate-competitive, with an IC_50_ value of 10 μM. Both compounds had good GSK-3β selectivity against a panel of ten other kinases. Using the computational tool GOLD 5.0, BTO-5s was confirmed to be substrate-competitive and therefore to bind in the substrate-binding pocket (Pocket 2, Figure 1), while BTO-5h binds to the same pocket as **6V** [37,94] (Pocket 7, Figure 1). In 2017, Gao et al., proposed a new family of compounds after modifying and optimizing the BTO-5h structure in order to replace the potential genotoxic hydrazine moiety [96]. They reported the synthesis of 23 new BTO derivatives with an IC_50_ value in the low micromolar range. Kinetics experiments demonstrated that a subset of these compounds (i.e., **20f** and **21f**, chosen as reference for the family, with IC_50_ values of 11.7 μM and 1.4 μM, respectively) were non-ATP/substrate-competitive GSK-3β inhibitors [96]. Another compound, **20g**, with the highest antitumoral activity in ovarian cancer cell lines, was chosen as representative compound to test the general selectivity and to undergo additional in vitro and in vivo analyses. The results showed that **20g** was not active against other kinases. Moreover, **20g** induced cell apoptosis, blocked the cell cycle in the G1 phase in different cancer cell lines, and reduced human xenograft tumor growth in nude mice [96]. These data are in agreement with GSK-3β’s regulatory role in the proliferation of ovarian cancer cells [97].

##### Compounds **4-3** and **4-4**

In 2020, the Eldar–Finkelman group published a novel class of small-molecule substrate-competitive GSK-3β inhibitors [103]. They proposed a computational analysis to screen and select possible small molecules mimicking the binding mode of a well-known substrate-competitive peptide inhibitor (L803). Based on the peptide model, the small molecules were designed to maintain specific pharmacophore properties and interactions with the substrate-binding pocket [104,105]. The L803 peptide pharmacophore model was obtained with Ligand 4.0, taking into consideration its key interactions with the protein. It was used for a virtual screening campaign of ~6.36 million compounds in the ZINC database (November 2020) against GSK-3β. The group selected and ranked the best compounds, using an iterative three-cycle virtual screening docking procedure associated with an in vitro ELISA-based kinase assay and a principal component analysis (PCA). They then designed and synthesized a novel class of inhibitors, starting from the hit compounds [103]. Compounds **4-3** and **4-4** were identified as the best SAR-driven synthetic compounds, with IC_50_ values of 1–4 μM. Their binding to the substrate pocket of GSK-3β was confirmed by performing an in vitro activity assay with both wild-type GSK-3β and F93A, an active GSK-3β mutant that does not bind the substrate or the reference peptide. Compound selectivity for GSK-3β (and GSK-3α) was assessed by testing them against a panel of 30 protein kinases. Finally, **4-4**′s biological effect was also tested by evaluating the β-catenin phosphorylation levels in neuroblastoma SH-SY5Y cells and in hippocampal primary neurons. As novel lead compounds for GSK-3β inhibition, **4-3** and **4-4** require further investigation for their potential development into novel drugs to treat different neurological pathologies [103].

##### VP0.7 and Derivatives

In 2011, starting from the computational investigation of the GSK-3β druggable pockets presented above, VP0.7 and its derivatives were identified as non-ATP-competitive GSK-3β inhibitors [37]. In an in vitro screening of an internal library of small molecules, the quinoline derivative N′-dodecanoyl-1-ethyl-4-hydroxy-2-oxo-1,2-dihydroquinoline-3-carbohydrazide VP0.7 showed GSK-3β inhibition, with an IC_50_ of 3.01 μM. In additional kinetics experiments, VP0.7 showed reversible non-ATP-competitive, non-substrate-competitive GSK-3β inhibition. To identify preferential binding sites for VP0.7 on the protein surface, researchers performed docking studies of VP0.7 on selected GSK-3β structures using Autodock software. These suggested that pocket 7 (Figure 1) is the preferential binding site for VP0.7 on GSK-3β. VP0.7 seems to orientate its aromatic ring (“sandwich”) between Arg209 and Thr235 and its aliphatic chain around the region determined by Leu169, Pro331, and Thr330. Notably, in mouse models of limb girdle muscular dystrophy R1 calpain 3-related (LGMDR1), in vivo administration of VP0.7 and Tideglusib restored the expression and phosphorylation of key proteins that are implicated in Wnt and mTOR signaling pathways and that are reduced in LGMDR1 patients. This suggests GSK-3β allosteric modulation could be a possible therapeutic strategy for this disease [98]. In addition, administration of VP0.7 and TDZD-8 in experimental mouse models of AE reduced pathology severity [99], hinting at GSK-3β as a feasible target for new therapeutic interventions for AE. The same group recently published a paper on the development and characterization of additional VP0.7 quinoline derivatives, with the aim of improving this compound family’s potency. Indeed, two of these newly developed inhibitors (compounds **53** and **59**) were confirmed to be noncompetitive for the substrate and ATP, with IC_50_ values of 2.01 μM and 2.48 μM, respectively [100]. The structural analysis ascribed the inhibitory allosteric effect of these quinoline derivatives to their ability to modify the flexibility of the GSK-3β activation loop, inducing a smooth conformational change in the active site. Finally, in in vitro survival studies, a positive effect of compound **53** was reported on cellular models of congenital myotonic dystrophy (CDM1) and spinal muscular atrophy (SMA) [100].

##### **6j** Squaramide

Recently, Carullo and colleagues proposed a new class of asymmetric squaramides as GSK-3β inhibitors [101]. This family of compounds was studied in vitro by testing their inhibitory effect on GSK-3β activity. Among these compounds, **6j** behaved as a non-ATP-competitive inhibitor, with an IC_50_ value of 3.5 μM. To further characterize this inhibitor, an in silico docking and molecular dynamics study was conducted. Compound **6j** was suggested to bind to the GSK-3β allosteric pocket 7, interacting with Arg209 (H-bond), His173 (π-π stacking and hydrophobic interaction), Leu207, and Val240 (hydrophobic interaction). An in silico evaluation of the physicochemical properties showed that this compound was optimal in terms of Lipinski’s rule of five, water solubility, and Caco-2 and BBB permeability. Finally, **6j** was tested for its toxicity and inhibition of GSK-3β in retinal pigment epithelium (RPE) cells. From these analyses, **6j** induced significant cell toxicity at 100 μM, and had significant inhibitory effects on the pathway downstream of GSK-3β at 2.5 μM [101]. These preliminary evaluations suggest **6j** is a candidate for future development as a novel allosteric GSK-3β inhibitor.

##### LCQFGS01 and LCQFGS02

Using an optimized virtual screening and docking procedure, the Silva group recently proposed an optimized pipeline to identify and characterize allosteric GSK-3β inhibitors and their most likely binding poses [102]. Briefly, the group used VP0.7 binding poses in GSK-3β’s allosteric pocket 7, confirmed by GLIDE software (Glide Schrödinger Suite; 2019-2), in order to perform a virtual screening campaign on the Chembridge CNS, eMolecule, and Princeton databases (including 13 million commercially available molecules). LCQFGS01 and LCQFGS02 were selected as promising binders of GSK-3β in allosteric pocket 7. Their in silico toxicological and pharmacokinetic profiles were improved relative to the reference compound VP0.7, despite maintaining similar binding interactions with the target protein. The docking predictions were corroborated and validated by quantum chemical energetic features’ calculation (i.e., the lowest unoccupied molecular orbital, LUMO and the highest occupied molecular orbital, HOMO). In vitro studies are needed to confirm this in silico work and the reliability of its predictions [102].

## 3. Discussion and Future Perspectives

Given the devastating effects of GSK-3β malfunctioning and/or altered expression in several pathologies, great efforts have been made to target this multifunctional protein. Most GSK-3β inhibitors belong to the large family of kinase inhibitors (KIs), which target the active site of kinases with low or no selectivity among the 500+ kinases of the human proteome. As discussed above, selectivity has always been a key issue to overcome in order to prevent side effects due to concurrent inhibition of other targets [106]. Moreover, because GSK-3β is involved in multiple pathways, completely inhibiting this target risks shutting down nonpathological pathways. A greater level of complexity is also due to the fact that each GSK-3β-related pathology is linked to the GSK-3β default in different pathways. Inhibitors must therefore be developed to target the specific pathology under consideration [107].

It is now clear that GSK-3β ATP competitive inhibitors are not the most suitable class of compounds for inhibiting this multifaceted and multifunctional target. Indeed, most ATP competitive inhibitors have failed preclinical trials due to toxicity and side effects.

Of the alternative GSK-3β inhibitors presented here, a peculiar subset are the irreversible inhibitors that covalently but selectively bind to the ATP-binding site (pocket 1). Their irreversible nature means that they completely inhibit the target, which is not always suitable for a target such as GSK-3β, which is involved in multiple pathways. Nevertheless, Tideglusib is the only GSK-3β ATP inhibitor to have reached clinical trials, albeit with the limitations described above (https://clinicaltrials.gov, accessed on 20 February 2023).

Substrate-binding compounds (pocket 2) bind a pocket that is less conserved among kinases than the orthosteric pocket. These compounds are more selective and less prone to inducing side effects than ATP-competitive inhibitors. Nevertheless, substrate-binding inhibitors have not been extensively studied. This is because they are usually difficult to design, with the substrate-binding site usually being poorly defined, large, and shallow. One example of this is palinurin and tricantin binding pocket 5, which is a shallow cavity that is difficult to target except with a long molecule such as the natural compounds here reported (i.e., palinurin and tricantin). Attempts have failed to identify small fragments that bind specific areas of the pocket. Pocket 7 appears to be the most promising site for the development of potential allosteric inhibitors. However, pockets 6 and 4 certainly merit further investigation, as suggested by Pocketron’s preliminary analysis.

One recent drug development approach to overcoming many challenges associated with GSK-3β is to search for allosteric druggable pockets. Allostery is a growing field. However, as discussed in this review, the search for allosteric GSK-3β inhibitors has not yet delivered significant improvements, relative to the size of research effort. The combination of computational analyses with biochemical and cell-based assays has successfully identified several allosteric compounds [37,102,106]. However, the efficacy of the resulting drugs is not yet significantly greater than that of ATP-competitive inhibitors. The turning point would be to identify novel ATP-alternative pockets that can host molecules capable of inducing conformational changes in GSK-3β that selectively impact its activity. Indeed, this review highlights how the search for allosteric GSK-3β inhibitors can be improved and developed further. There are drug discovery approaches that have barely been applied to this target, and there are pockets that have not yet been fully characterized by advanced computational and drug discovery approaches.

A fragment-based approach, for example, has rarely been used to identify allosteric GSK-3β inhibitors [108]. Such an approach would allow a punctual and complete pocket occupation, which is more suitable for identifying critical areas within broad and shallow pockets. Recently, a similar approach was successfully applied to another kinase, i.e., dual specificity mitogen-activated protein kinase 1 (MEK1) [109]. We have here shown how the use of advanced computational analyses to identify critical crosstalk between the orthosteric site and alternative pockets can be a valid method of seeking potential allosteric pockets. The preliminary analysis pinpointed pocket 4 as an interesting extended channel directly connected to the orthosteric site. Furthermore, the authors identified additional pockets that were not previously observed.

To improve and develop the promising field of GSK-3β allosteric inhibitors, concerted computational and biophysical efforts are needed to reveal dynamical and long-range correlations. Structural and molecular dynamics studies should work in parallel to shed light on the plasticity and mobility of the GSK-3β pockets, so that researchers can understand why they have been so hard to target [107,110]. There are two key potential consequences of the crosstalk and interconnection between different binding sites. First, allosteric pockets could be extremely mobile. Second, an inhibitor could bind a given allosteric site in only one specific ligand-bound/unbound state of GSK-3β. Protein dynamics may be the key to identifying effective allosteric GSK-3β inhibitors. Indeed, for other targets, achievements in 3D structural biology have been critical to identifying and investigating new allosteric mechanisms. While several structures are available for GSK-3β alone or in complex with ATP or with substrate-competitive inhibitors, no 3D structures are available for this target in complex with the potentially allosteric inhibitors identified to date. This suggests that the peculiar dynamic arrangement of allosteric binding is a barrier to achieving an atomic-resolution 3D structure of the complex. Indeed, drug designers and developers should carefully consider the protein dynamics when searching for novel and selective inhibitors of GSK-3β. This also applies to other kinases. Advanced computational approaches to investigating protein dynamics would thus have a significant impact on the search for selective and efficient allosteric GSK-3β inhibitors. The identification of potential allosteric binding sites would then require experimental validation of whether a drug-like molecule binding to one of these pockets can actually modulate the protein’s activity.

In this review, we have provided an updated overview of the available non-ATP-competitive GSK-3β inhibitors. We have thus highlighted the continuing value of the allosteric approach, which may be the key to identifying effective and selective GSK-3β inhibitors.

## Figures and Tables

**Table 1:** Key residues of the main GSK-3β pockets (indicated as P1–P7), as described in Palomo et al., 2011 [37]. The residue’s numbering is also reported according to PDB 1J1C, chain B.

	GSK-3β Pocket	Pocket Key Residues									
Numbering of Palomo et al.	**P1**	85	97	113	134	135	138	186	188	199	200
Residue’s identity		Lys	Glu	Asp	Tyr	Val	Thr	Asn	Leu	Cys	Asp
1J1C_B numbering		563	575	591	612	613	616	664	666	677	678
Numbering of Palomo et al.	**P2**	67	89	94	93	95	96	97	180	205	
Residue’s identity		Phe	Gln	Lys	Phe	Asn	Arg	Glu	Arg	Lys	
1J1C_B numbering		545	567	572	571	573	574	575	658	683	
Numbering of Palomo et al.	**P3**	215	220	223	229						
Residue’s identity		Ser	Arg	Arg	Phe						
1J1C_B numbering		693	698	701	707						
Numbering of Palomo et al.	**P4**	140	144	148	185	219	220	221	222	249	
Residue’s identity		Tyr	Arg	Arg	Gln	Ser	Arg	Tyr	Tyr	Glu	
1J1C_B numbering		618	622	626	663	697	698	699	700	727	
Numbering of Palomo et al.	**P5**	26	38	56	71	86	119				
Residue’s identity		Met	Thr	Tyr	Tyr	Lys	Ser				
1J1C_B numbering		504	516	534	549	564	597				
Numbering of Palomo et al.	**P6**	80	111	113	133	135	190	197			
Residue’s identity		Glu	Arg	Arg	Asp	Val	Asp	Lys			
1J1C_B numbering		558	589	591	611	613	668	675			
Numbering of Palomo et al.	**P7**	173	178	207	209	211	234	235	236	330	369
Residue’s identity		His	Cys	Leu	Arg	Glu	Tyr	Thr	Ser	Thr	Ser
1J1C_B numbering		651	656	685	687	689	712	713	714	808	847

List of abbreviations: Lys (Lysine); Glu (Glutamate); Asp (Aspartate); Tyr (Tyrosine); Val (Valine); Thr (Threonine); Asn (Asparagine); Leu (Leucine); Cys (Cysteine); Phe (Phenylalanine); Gln (Glutamine); Ser (Serine); Met (Methionine); Arg (Arginine); His (Histidine).

**Table 2:** Allosteric and non-ATP-competitive GSK-3β inhibitors.

Inhibitor	Potency (Kd/IC_50_)	In Vitro Assays	In Cellulo/Ex Vivo Assays	In Vivo/Clinical Trial	Pocket (Docking)	Preclinical Models	Ref.
Palinurin Tricantin	4.5 μM 7.5 μM	^33^P-γ-ATP-based GSK-3β inhibition assay	Inhibition of tau phosphorylation; cell viability	-	Pocket 5	AD	[42,43,44,45,46,47]
Manzamine A	10.0 μM	^33^P-γ-ATP-based GSK-3β inhibition assay	Inhibition of tau phosphorylation; cell viability, proliferation and invasion; apoptosis induction; anti-neuroinflammatory activity	Pharmacokinetic studies; tumor growth inhibition	Pocket 2 (substrate pocket)	AD, neuroinflammation, PaC, PC and CRC	[48,49,50,51,52,53,54,55,56]
TDZD-8	2.0 μM	^32^P-γ-ATP-based GSK-3β inhibition assay	p53 induction; apoptosis analysis; cell viability, toxicity and proliferation; oxidative stress evaluation; inhibition of tau phosphorylation;	Locomotor activity; behavioral studies; attention deficits; information processing; ischemic-induced brain injury; apoptosis suppression; neuronal protection; tumor growth inhibition; effect on joint injury; inflammation effect	Pocket 1 (ATP pocket)	AD, CRC, glioblastoma, PC, PD, OC, IBI, leukemia, schizophrenia, type II diabetes, RA, colitis	[57,58,59,60,61,62,63,64,65,66,67,68,69,70,71,72,73,74,75,76,77]
Tideglusib	-	FRET-based GSK-3β inhibition assay; GSK-3β reversibility inhibition studies; ^35^S-tideglusib GSK-3β interaction assay	-	ARGO Phase II clinical trial; TAUROS Phase II clinical trial; escalating dose pilot trial	Pocket 1 (ATP pocket)	-	[78,79,80,81,82,83]
HMK-32	1.5 μM	^32^P-γ-ATP-GSK-3β inhibition assay; ^32^P-γ-ATP-based GSK-3β reversibility inhibition studies	Inhibition of tau phosphorylation; neuritogenesis evaluation	-	Pocket 1 (ATP pocket)	AD	[84,85,86]
ITDZ (VP3.15)	0.9 μM	Luminescence-based GSK-3β inhibition assay; GSK-3β enzymatic inhibition; luminescence-based GSK-3β reversibility studies	Neuroprotection and neurogenesis assessment; BBB penetration; cell survival, proliferation and differentiation; ex vivo remyelination	Remyelination; pharmacokinetic studies; inflammatory and degenerative signs; visual function; EAE clinical signs examination	Pocket 2 (Substrate Pocket)	LGMD, AE, MS, RP	[87,88,89,90,91,92,93]
BTO (20f, 20g)	11.7 μM 22.4 μM	Luminescence-based GSK-3β inhibition assay	Cell viability; cell cycle analysis; apoptosis induction	Tumor growth inhibition	Pocket 2 (Substrate Pocket)	OC	[94,95,96,97]
4-3 4-4	1.0–4.0 μM	ELISA-based GSK-3β inhibition assay	Wnt/β-catenin pathway evaluation	-	Pocket 2 (Substrate Pocket)	AD	[98,99,100]
VP0.7	3.0 μM	Luminescence-based GSK-3β inhibition assay	Wnt/β-catenin pathway evaluation	EAE clinical signs examination	Pocket 7	AE	[37,98,99,100]
6j	3.5 μM	FRET-based GSK-3β inhibition assay	Cell viability; β-catenin-mediated TCF/LEF transcriptional activity	-	Pocket 7	Retinal epithelium	[101]
LCQFGS01 LCQFGS02	-	-	-	-	Pocket 7	In silico evaluation only	[102]

List of abbreviations: AE (autoimmune encephalomyelitis); ARGO (a phase II study of atezolizumab with rituximab, gemcitabine, and oxaliplatin); BBB (blood–brain barrier); CRC (colorectal cancer); EAE (experimental autoimmune encephalomyelitis); ELISA (enzyme-linked immunosorbent assay); FRET (fluorescence resonance energy transfer); IBI (ischemic brain injury); LGMD (limb girdle muscular dystrophy); MS (multiple sclerosis); OC (ovarian cancer); PaC (pancreatic cancer); PC (prostate cancer); RA (rheumatoid arthritis); RP (retinitis pigmentosa); TAUROS (tau restoration on PSP study); TCF/LEF (T cell factor/lymphoid enhancer factor family).

## Data Availability

Not applicable.
